# The Impact of COVID-19 on Routine Medical Care and Cancer Screening

**DOI:** 10.1007/s11606-021-07254-x

**Published:** 2022-01-10

**Authors:** Neil S. Wenger, Annette L. Stanton, Ryan Baxter-King, Karen Sepucha, Lynn Vavreck, Arash Naeim

**Affiliations:** 1 Division of General Internal Medicine and Health Sciences Research, David Geffen School of Medicine at UCLA, 1100 Glendon Avenue #804, Los Angeles, CA 90024 USA; 2 Departments of Psychology and Psychiatry/Biobehavioral Sciences, UCLA, Los Angeles, CA USA; 3 Department of Political Science, UCLA College, Los Angeles, CA USA; 4 Health Decision Sciences Center, Massachusetts General Hospital, Harvard Medical School, Boston, MA USA; 5 Departments of Political Science and Communication, UCLA College, Los Angeles, CA USA; 6 UCLA Center for SMART Health, David Geffen School of Medicine at UCLA, Los Angeles, CA USA

**Keywords:** COVID-19, missed medical appointments, cancer screening

## Abstract

**Background:**

COVID-19 restrictions and fear dramatically changed the use of medical care. Understanding the magnitude of cancelled and postponed appointments and associated factors can help identify approaches to mitigate unmet need.

**Objective:**

To determine the proportion of medical visits cancelled or postponed and for whom. We hypothesized that adults with serious medical conditions and those with higher anxiety, depressive symptoms, and avoidance-oriented coping would have more cancellations/postponements.

**Design:**

Four nationally representative cross-sectional surveys conducted online in May, July, October, and December 2020.

**Participants:**

59,747 US adults who completed 15-min online surveys. 69% cooperation rate.

**Measures:**

Physical and mental health visits and cancer screening cancelled or postponed over prior 2 months. Plan to cancel or postpone visits over the next 2 months. Relationship with demographics, medical conditions, local COVID-19 death rate, anxiety, depressive symptoms, coping, intolerance of uncertainty, and perceived COVID-19 risk.

**Key Results:**

Of the 58% (*N* = 34,868) with a medical appointment during the 2 months before the survey, 64% had an appointment cancelled or postponed in May, decreasing to 37% in December. Of the 41% of respondents with scheduled cancer screening, 20% cancelled/postponed, which was stable May to December. People with more medical conditions were more likely to cancel or postpone medical visits (OR 1.19 per condition, 95% CI 1.16, 1.22) and cancer screening (OR 1.20, 95% CI 1.15, 1.24). Race, ethnicity, and income had weak associations with cancelled/postponed visits, local death rate was unrelated, but anxiety and depressive symptoms were strongly related to cancellations, and this grew between May and December.

**Conclusions:**

Cancelled medical care and cancer screening were more common among persons with medical conditions, anxiety and depression, even after accounting for COVID-19 deaths. Outreach and support to ensure that patients are not avoiding needed care due to anxiety, depression and inaccurate perceptions of risk will be important.

**Supplementary Information:**

The online version contains supplementary material available at 10.1007/s11606-021-07254-x.

## INTRODUCTION

COVID-19 has upended myriad aspects of life, particularly receipt of usual medical care. Early in the pandemic, many health care providers cancelled elective procedures and visits^1^ and medical visits were frequently avoided.^2^ These actions were logical because medical facilities are where COVID-19-infected patients are diagnosed and treated, medical interactions often violate social distancing, and people needing medical care often are at risk for adverse outcomes from COVID-19. Prior reports suggest that 20–50% of adults had cancellations of medical care^3,4^ with declines in emergency services^5,6^ and more cancellations for those with pre-existing medical conditions.^3^ Studies show substantial decreases in admissions for serious medical conditions.^7,8^

Increased levels of non-COVID-19 death have been reported during these periods of medical care avoidance.^9^ Early data suggest fewer early cancer diagnoses^10^ and portend worse outcomes.^11,12^ Further, missed appointments and treatments among patients with existent cancer are expected to result in increased cancer mortality.^13^

To plan for medical care as restrictions ease, it is important to understand the developing level of unmet need for clinical care, trends over time, and patient characteristics associated with cancelled or postponed medical appointments and cancer screening. We surveyed cross-sectional national samples at four time points in 2020 to examine missed medical care and cancer screening in the setting of COVID-19. We hypothesized that in addition to the local death rate from COVID-19, people at greater risk of adverse outcomes from COVID-19, those with higher anxiety and depressive symptoms, those more intolerant of uncertainty, and those more likely to cope with the pandemic through avoidance would be more likely to have cancelled and postponed medical care including cancer screenings.

## METHODS

We fielded four 15,000-person cross-sectional surveys between May and December of 2020. Surveys were conducted online with samples provided by the market research firm Lucid. Samples are constructed to match a set of demographic quotas on age, gender, race, ethnicity, region, income, and education. The resulting survey data are weighted to be representative of the US adult population (eTable 1) as described in the Appendix. The response rate was 69%. This project was approved by the UCLA Institutional Review Board (IRB #20-000786).

### Outcome Variables

The survey asked about missed medical appointments as follows: “Over the last two months, have any of your healthcare providers cancelled or postponed scheduled visits or services for physical or mental health?” Response options were Yes, No, I did not have anything scheduled, and Not sure. Respondents also were asked if they had cancelled or postponed scheduled visits or services. These two items were combined to describe whether the respondent had a medical visit that was cancelled or postponed, had no appointment scheduled, attended a medical visit, or was unsure. Concerning cancer screening, the survey asked: “Over the last two months, have you cancelled or postponed getting routine cancer screening (breast cancer mammography, colonoscopy, etc.)?” with the same response options. Concerning the future, respondents were asked “In the upcoming two months, do you plan to cancel or postpone getting routine cancer screening (breast cancer mammography, colonoscopy, etc.)?” and the same question was asked concerning visits or services for physical or mental health.

### Key Independent Variables

Independent variables included age, gender, race/ethnicity, education, household income (by tercile), political party, number of significant medical diagnoses (heart disease, lung disease, diabetes, hypertension, cancer, other), respondent or household member believes they had COVID-19, coping with “the COVID-19 experience” (yes/no items based on commonly used measures)^14,15^ divided into approach-oriented coping (i.e., active coping attempts to acknowledge the stressor and promote care of self/others [e.g., “accepting the reality,” “pursuing creative activities”]) by tercile and avoidant coping (e.g., “sleeping more than usual to think about it less,” “pretending it hasn’t really happened”) dichotomized, anxiety,^16^ depressive symptoms,^17^ three items from the Intolerance of Uncertainty Scale,^18^ perceived COVID-19 risk, region, and time (survey wave). COVID-19 deaths in the county of the respondent 2 weeks before each survey were obtained from nytimes/covid-19-data, https://github.com/nytimes/covid-19-data/tree/master/excess-deaths.

### Analysis

The data include 14,636 interviews conducted in May 11–24, 14,936 interviews in July 9–22, 14,946 interviews in October 1–17, and 15,229 interviews in December 4–16. Weighted proportions (Table 1) were calculated with R statistical software, version 3.6.1. Weighted multivariate logistic regression was used to calculate odds ratios for each of the medical visit variables. This was performed independently for each survey wave and overall with survey wave included as a fixed effect.

**Table 1:** Characteristics of Survey Respondents

Variable	Level	Weighted %
Age, years	18–39^a^	33.4%
	40–64	43.7%
	65+	22.8%
Gender	Male^a^	47.0%
	Female	53.0%
Education	High school or less^a^	29.2%
	Some college	37.3%
	College and above	33.5%
Household income	$34,999 or less^a^	19.1%
	$35,000–$79,999	34.8%
	$80,000 or more	46.1%
	Missing	4.4%
Political party identification	Democrat^a^	46.3%
	Independent	15.9%
	Republican	37.8%
COVID-19 infection in past 2 months	No infection for self or household^a^	88.0%
	Tested positive for COVID-19	4.1%
	Believes had COVID-19 but no positive test	5.7%
	Believes household had COVID-19, but not self	2.2%
Race/ethnicity	White^a^	65.2%
	Black or African American	11.3%
	Hispanic	14.9%
	Asian or Pacific Islander	5.8%
	Other race	2.8%
Region	Northeast^a^	17.9%
	Midwest	20.9%
	South	38.3%
	West	22.9%
Depression	No depression^a^	51.3%
	Mild depression	23.7%
	Moderate depression	13.9%
	Moderately severe depression	7.0%
	Severe depression	4.1%
Perceived COVID-19 risk in the next 30 days	COVID-19 risk: very low^a^	32.6%
	COVID-19 risk: moderately low	27.4%
	COVID-19 risk: neither high nor low	27.2%
	COVID-19 risk: moderately high	9.7%
	COVID-19 risk: very high	2.9%
Anxiety level, past 7 days	Normal anxiety^a^	42.0%
	Mild anxiety	23.9%
	Moderate anxiety	26.2%
	Severe anxiety	7.9%
Uncertainty tolerance	High uncertainty tolerance^a^	40.3%
	Medium uncertainty tolerance	36.2%
	Low uncertainty tolerance	23.5%
Coping (approach)	Coping, approach low^a^	36.3%
	Coping, approach moderate	40.2%
	Coping, approach high	23.5%
Coping (avoid)	Coping, no avoidance (no behaviors)^a^	59.2%
	Coping, avoidance (any behaviors)	40.8%
Survey wave	Wave 1^a^	23.1%
	Wave 2	24.3%
	Wave 3	25.2%
	Wave 4	27.4%
# of medical conditions	0^a^	48.2%
	1	31.8%
	2	13.6%
	3	4.6%
	4	1.3%
	5	0.4%
	6	0.1%

^a^Referent category for regression presented in Figure 2 and eFigure 1

## RESULTS

Overall, 58% (*N* = 34,868) of the respondents had a medical appointment during the 2 months prior to survey, and this increased slightly from May through December. Of the people with a physical or mental health appointment, 51% (17,836) did not attend because the appointment was cancelled or postponed. This fraction decreased across the first three waves from 64.6% in May to 53.5% in July and 36.8% in October, before stabilizing at 37.6% in December (Table 2). The first few months of the pandemic were especially severe, with nearly two-thirds of respondents reporting a missed appointment in the 2 months immediately after lockdowns were put in place and more than half reporting missed visits through the summer.

**Table 2:** Cancelled or Postponed Physical and Mental Health Care and Cancer Screening over 1 Year of Pandemic

	Mar 2020–May 2020	May 2020–July 2020	July 2020–Oct 2020	Oct 2020–Dec 2020	Dec 2020–Feb 2021
Cancelled or postponed physical and mental health care					
Cancelled or postponed physical or mental health appointment in the past 2 months by the patient or provider	64.6%	53.5%	36.8%	37.6%	
Patient plans cancellation or postponement of physical or mental health appointment in the next 2 months		17.4%	15.2%	13.0%	14.0%
Cancelled or postponed cancer screening					
Cancelled or postponed cancer screening in the past 2 months by the patient	19.8%	20.5%	19.1%	19.4%	
Patient plans cancellation or postponement of cancer screening in the next 2 months		15.0%	16.2%	15.6%	17.1%

“Not sure” and “Nothing scheduled” responses excluded. Note that respondents reported cancellations and postponements for the past 2 months and planned cancellation and postponements over the next 2 months. Thus, comparisons between past and future within a time period are between two different survey wave cohorts. Surveys were conducted May 11 to 24, July 9 to 22, October 1 to 17 and December 4 to 16 in 2020

Forty-one percent of respondents had a scheduled cancer screening in the 2 months prior to their survey, 56% had no cancer screening scheduled, and 3% were unsure. Of the 41% with a scheduled cancer screening, 19.7% cancelled or postponed (8% of the total survey population). The proportion of respondents with cancer screening appointments increased steadily across the four waves—indicating people’s return to activities over the course of the year—from 36.4% in May to 47.0% (*p* < 0.001) in December. The nearly 20% cancellation rate of retrospective cancer screening was stable across survey waves (Table 2).

Concerning future medical care, 50% of respondents reported having an appointment scheduled in the 2 months after their survey (6% unsure). Of those with appointments, 15% planned to cancel or postpone. Similarly, concerning future cancer screening, 42% had screening scheduled (50% no appointment, 8% unsure) and 16% (8.5% of the total survey population) planned to cancel or postpone.

Comparing reports of past appointment cancellations or postponements across survey waves to plans to cancel or postpone future appointments shows that retrospective reports far exceed anticipated cancellations or postponements for general medical care, whereas the difference between past and future cancellations or postponements is smaller for cancer screening. While physical and mental health appointment cancellations or postponements decreased and then plateaued, cancer screening cancellations were stable. Anticipated cancellations or postponements were stable for physical and mental health appointments and also cancer screening (Table 2).

### Medical Conditions and Missed Appointments

**Figure 1:**
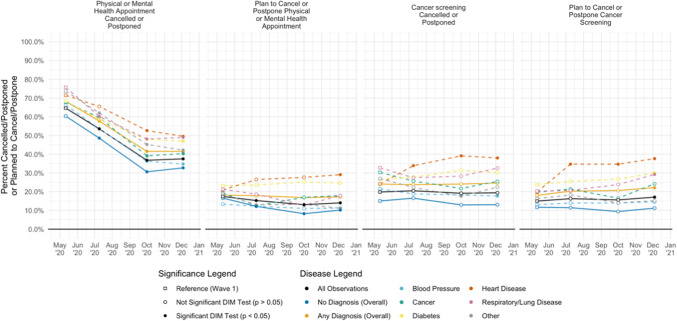
Physical and mental health appointments cancelled or postponed and cancer screening cancelled or postponed, overall and by medical condition. Columns 1 and 3 refer to cancellations or postponements in the past 2 months. Columns 2 and 4 refer to plans to cancel or postpone in the next 2 months. “Nothing scheduled” and “Not sure” excluded from analysis. DIM test = weighted difference-in-means test.

Overall, 45% of respondents reported having one or more of the serious medical conditions about which we asked: hypertension 27%, diabetes 15%, heart disease 7%, lung disease 6%, cancer 3%, and other 11%. Respondents with at least one medical condition (compared to those without any) were more likely to have a scheduled appointment within the 2 months prior to their interview (69% v 47%, *p* < 0.001). Those with an appointment and with medical conditions were more likely to experience a cancellation or postponement relative to those with appointments and no medical conditions (52% v 43%, 95% CI for difference 7.1–10.4%, *p* < 0.001). Patients with pre-existing heart and lung disease experienced the highest rates of cancelled or postponed appointments (Fig. 1).

Twenty-four percent of patients with serious medical conditions cancelled or postponed cancer screening or had these tests cancelled by providers, which was consistent across survey waves, compared to a 14% cancellation rate for respondents without such medical conditions (95% CI for difference 8.4–11.3%, *p* < 0.001) (Fig. 1).

Respondents with medical conditions compared to those without increasingly planned to cancel or postpone future physical and mental health appointments (17% v 12%, 95% CI for difference 4.6–7.2%, *p* < 0.001) and cancer screenings (20% v 11%, 95% CI for difference 8.2–10.9, *p* < 0.001) (Fig. 1).

### Local COVID-19 Death Rate

**Figure 2:**
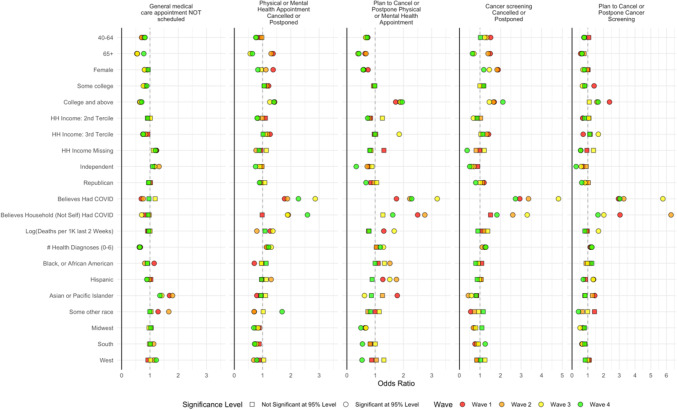

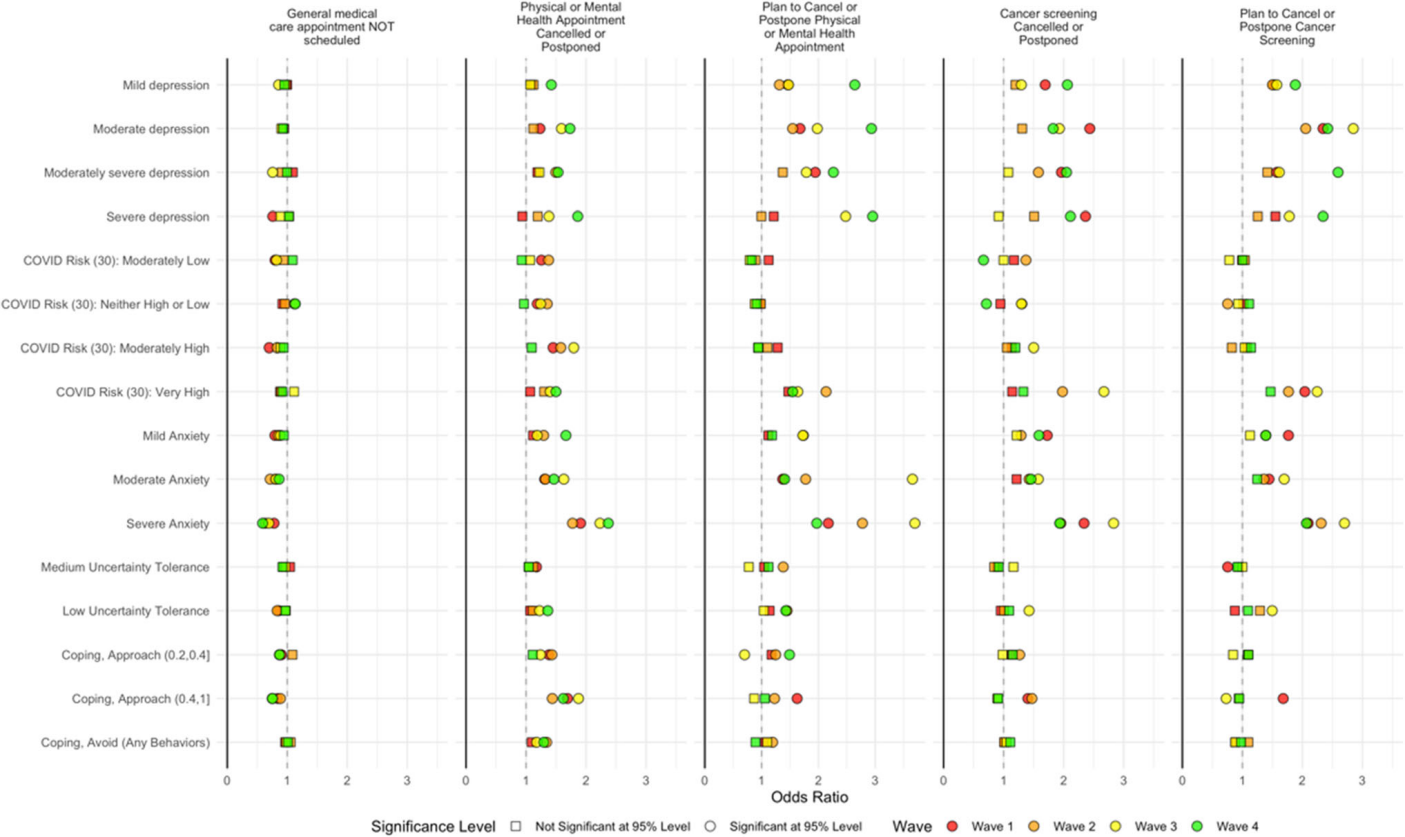
Factors associated with cancelled and postponed physical and mental health care and cancer screening, May to December. Separate regression models for each survey wave. For referent categories, see Table 1. Results control for variables in Table 1. *N* varies by model. Note that the *x*-axis varies by column. For columns 2–5, “Nothing scheduled” excluded from analysis. Dependent variable equals 1 if care is scheduled and cancelled or postponed.

The local death rate from COVID-19 was unrelated overall in adjusted analyses with appointment cancellation or postponement (eTable 2). As seen in Figure 2, across time points in adjusted analyses from May to December, the local COVID-19 death rate had minimal relationship with past or future physical or mental health appointments or cancer screening.

### Characteristics of People with Cancelled/Postponed Care

#### Routine Care: Past Cancellations

Adjusted analyses for the four cancellation/postponement variables are displayed by survey wave in Figure 2(and with all survey waves combined in eTable 2 and eFigure 1). Concerning physical and mental health appointments, visits were more often cancelled or postponed for women and those in the highest tercile of income in the first survey wave, but in later waves, gender and income were less strongly related to cancellations. Race/ethnicity was not significantly related to cancellation in most survey waves. In early survey waves, appointments were more likely to be cancelled/postponed by or for respondents 65 years and older than people under 30, but cancellation/postponement was significantly less likely for this group in October and December. Compared to people with a high school education or less, college graduates were more likely to experience appointments cancelled/postponed. This was also the case for respondents who believed that they or someone in their household had COVID-19 and those with higher perceived risk of COVID-19. By December, only a very high level of perceived risk of COVID-19 was associated with cancelled/postponed appointments. Each medical condition was associated with 1.19 odds of cancelling/postponing an appointment (95% CI 1.16–1.22), and this association was consistent across survey waves. Higher levels of anxiety and symptoms of depression were associated with cancelled/postponed appointments, particularly during December. Low tolerance of uncertainty was associated with cancelled appointments during survey waves 3 and 4. Appointment cancellation/postponement was more common for respondents reporting avoidance-oriented coping and also for those with more approach-oriented coping strategies (Fig. 2).

#### Routine Care: Plans to Cancel

Factors associated with cancelling future physical and mental health care were similar to those associated with past cancelled visits, with college graduates more likely to plan to cancel appointments and a minimal relationship with race/ethnicity and household income except that Hispanic ethnicity was associated with an increased likelihood of cancellation during early survey waves, but not in December. Number of medical conditions was statistically significantly related to future cancellation in the latter two survey waves. Personal or family member infection with COVID-19 was strongly related to cancelling future appointments; otherwise, perceived COVID-19 risk played a minimal role in planned cancellation. Anxiety and depressive symptoms played a much larger role: depressive symptoms were strongly associated with cancelling future appointments, particularly in December; the relationship with anxiety seemed to peak in October. Coping and uncertainty tolerance were largely unrelated to future cancellation plans (Fig. 2).

#### Cancer Screening: Past Cancellations

Cancer screening was cancelled more often by women and college graduates. As seen for general medical care, people 65 years and older were more likely to cancel in early survey waves and less likely in October and December. Race/ethnicity, income, and perceived risk of COVID-19 had minimal relationships with cancelled cancer screening although respondents who identified as Asian or as a Pacific Islander were less likely to cancel screening than those identifying as white during survey waves 2 and 3. Much like general medical care cancellations, people with greater anxiety and depressive symptoms were more likely to cancel cancer screening. Approach-oriented coping was strongly related to cancelled cancer screening during early waves but not in October or December, and avoidant coping was not associated significantly with cancellations (Fig. 2).

#### Cancer Screening: Plans to Cancel

Cancelling/postponing future cancer screening was related to factors similar to cancelling/postponing future physical and mental health appointments with older people and women less likely, college graduates more likely and minimal relationships with race/ethnicity and income. Severe anxiety was associated with planning to cancel cancer screening, and the relationship with level of depressive symptoms was large, particularly in latter survey waves.

## DISCUSSION

More than half of the respondents in a nationwide representative sample of nearly 60,000 US adults had scheduled medical care appointments cancelled or postponed from March to December 2020. People with more serious medical conditions were more likely to have cancelled care. Many of these visits were likely for chronic disease management, and while some of these issues can be addressed via telemedicine, which dramatically increased during this time,^19^ distant interaction is likely inadequate for care that requires a physical exam or depends on laboratory testing. A survey in May 2000 found that one in five people who missed medical appointments due to COVID-19 reported that their condition worsened.^20^ The good news is that typically underserved groups—those with lower education and lower income and minority groups—were not more likely to have cancelled/postponed medical visits after accounting for other factors. Instead, anxiety, depressive symptoms, perceived COVID-19 risk and pandemic-related coping appear to be more strongly associated with appointment cancellations. These factors remain strong predictors of cancelled and missed care even accounting for local COVID-19 death rate in the surrounding area. Just because deaths are declining and hospitals are decompressing does not mean the chronically ill patient with severe anxiety will be seeking care. These findings might guide targeting of patient groups for catch-up care.

Anxiety and depression are far more prevalent in the setting of COVID-19,^21^ and these conditions may be affecting those without the condition in the past even more than those with pre-existing anxiety and depression.^22^ The relationship between greater depressive symptoms and more cancellations was most pronounced in December 2020 and particularly for future appointment cancellations. A similar pattern was seen for severe anxiety. Targeted accommodations and outreach to patients with anxiety and depression, which may be new conditions prompted by the pandemic experience and not previously known to the clinician, may be needed to restore medical follow-up and cancer screening.^22^

These findings expand on delayed or avoided medical care as described in the CDC June 2020 survey.^3^ That survey found similar relationships of missed care with demographic characteristics and also reported more missed visits among those with underlying medical conditions. This report adds by covering the pandemic period through 2020, considering specific medical conditions and including psychosocial measures, which have large associations with cancelled appointments.

About one-fifth of scheduled cancer screening was cancelled or postponed between March and December. Women, those over 40 years of age, and particularly those 65 years and older early in the pandemic, were more likely to have cancelled cancer screening in the prior 2 months, but not future appointments, suggesting a plan for catch-up, which is reasonable given that cancer screening is episodic with a frequency often measured in years. On the other hand, people with pre-existing medical conditions and those with depressive symptoms and anxiety were more likely to cancel past and future cancer screening, even after adjusting for perceived COVID-19 risk. Attention is needed to ensure that these individuals receive missed mammograms, pap smears, colonoscopies and other cancer screening.

This study has several limitations. Though each wave of the sample is representative of the nation, respondents are not impaneled across waves; thus, changes over time are averages and not within-person differences. In addition, missed visits and cancer screenings were self-reported, which might underestimate actual cancellations. Furthermore, we are unable to distinguish physical from mental health visits and also do not know whether visits occurred in person or remotely. Finally, interpretation of these data should consider the possibility that anxiety and depression may be the effects of cancellations as well as causes of cancellations.

These data provide insight into the enormous burden of missed medical care and prevention that health systems and individual clinicians must help patients to recoup. They identify anxiety and depression as key obstacles to be addressed and that cancelled/postponed care is most problematic for those who can least afford it: individuals with underlying medical conditions.

## Supplementary Information

ESM 1 (PDF 547 kb)

ESM 2 (PDF 489 kb)
