# Supplementary material for: The Impact of COVID-19 on Routine Medical Care and Cancer Screening
Source: J Gen Intern Med. 2022 Jan 10;37(6):1450–6. doi: 10.1007/s11606-021-07254-x (PMC8744580; doi:10.1007/s11606-021-07254-x)

eFigure1. Factors Associated with Cancelled and Postponed Physical and Mental Health Care and Cancer Screening, All Survey Waves

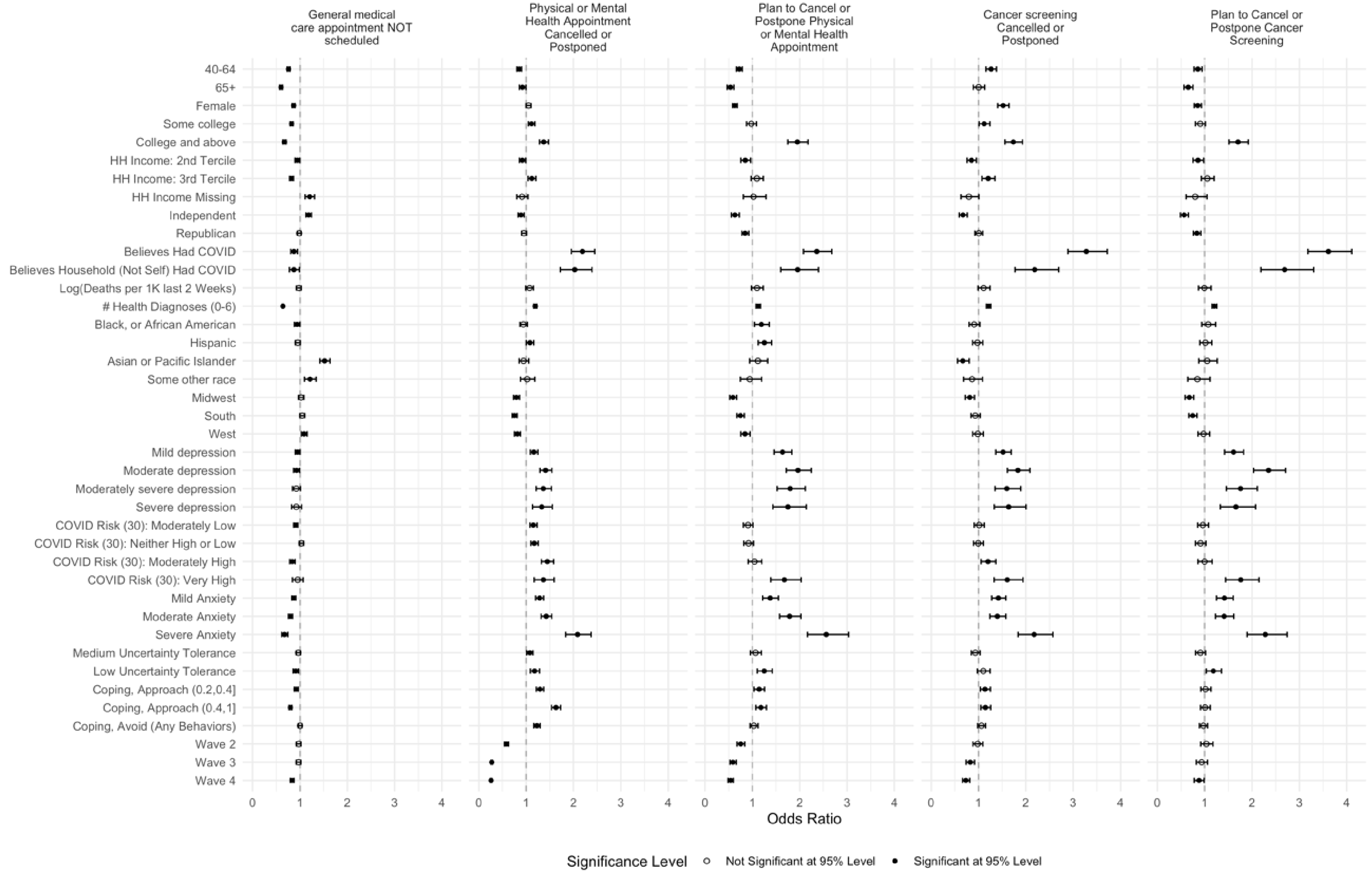

Supplement: Supplementary file 1 — (PDF 547 kb) [file 11606_2021_7254_MOESM1_ESM.pdf]
