# Supplementary material for: The Impact of COVID-19 on Routine Medical Care and Cancer Screening
Source: J Gen Intern Med. 2022 Jan 10;37(6):1450–6. doi: 10.1007/s11606-021-07254-x (PMC8744580; doi:10.1007/s11606-021-07254-x)
Supplement: Supplementary file 2 — (PDF 489 kb) [file 11606_2021_7254_MOESM2_ESM.pdf]

Online Supplemental Appendix: Derivation of Study Sample

As part of the UCLA COVID Health and Politics Project, we fielded four 15,000-person cross-sectional surveys between May and December of 2020. Surveys were conducted online with sample provided by the market research firm Lucid. Samples were constructed to match a set of demographic quotas on age, gender, race, ethnicity, region, income, and education. The resulting survey data are weighted to be representative of the U.S. adult population. The targets to which the data are weighted are derived from the U.S. adult population of the 2017 American Community Survey of the U.S. Census Bureau. We weight on the following factors: gender, the four major census regions, race, Hispanic ethnicity, household income, education, age, language spoken at home, nativity (U.S.- or foreign-born), 2016 presidential vote, and the urban-rural mix of the respondent's ZIP Code. We also weight on the following interactions: Hispanic ethnicity by language spoken at home, education by gender, gender by race, race by Hispanic origin, race by education, and Hispanic origin by education.

Nearly all contemporary surveys lack the properties of random selection, even those that derive from random sampling procedures, due to low response rates in the population.[1] The combination of quotas (used to ensure balance in the sample's composition) and post-stratification weights (used to ensure balance across sub-groups) return samples that are representative of the U.S. population with respect to the set of targets we identify with a high rate of accuracy. In 2016 and 2018, the Pew Research Center compared estimates generated by several online surveys to estimates of the same quantities in U.S. Census data and other high-quality sources, primarily the American Community Survey conducted by the U.S. Census Bureau and supplements to the Census Bureau's Current Population Study. We conducted the same assessments using the sampling approach and survey methods used in the UCLA COVID-

19 Health and Politics Study. For the various online samples analyzed by Pew, the median absolute difference from the government survey benchmarks was 3.6 percent. For the same set of survey questions, the median absolute difference between our methods and the government survey benchmarks was slightly lower (3.4%). More detail concerning survey procedures and these assessments is available.[2]

References:

1. Kennedy C, Deane C. What our transition to online polling means for decades of phone survey trends. https://www.pewresearch.org/fact-tank/2019/02/27/what-our-transition-to-online-polling-means-for-decades-of-phone-survey-trends/

2. Tausanovitch C, Vavreck L, Reny T, Hayes AR, Rudkin A. The Democracy Fund + UCLA Nationscape Methodology and Representativeness Assessment. 2019. Available at: https://www.voterstudygroup.org/uploads/reports/Data/NS-Methodology-Representativeness-Assessment.pdf. Accessed February 17, 2021.

Online Supplemental Appendix Table 1: Representativeness of Survey Sample

Variable	Value	Target %	Unweighted N	Weighted %
Age	18–23	9.4	4289	9.4
	24–29	10.9	4428	10.9
	30–39	17.4	8930	17.4
	40–49	16.3	9256	16.3
	50–59	17	7645	17
	60–69	15.2	6799	15.1
	70+	13.8	3171	13.8
Education	No high school diploma	11.6	4276	11.5
	High school diploma	27.3	9435	27.3
	Some college	21.9	7974	21.9
	Associate’s degree	8.4	4326	8.4
	Bachelor’s degree	19.3	12353	19.3
	Graduate degree	11.5	6154	11.5
Gender	Male	48.3	20449	48.3
	Female	51.7	24069	51.7
Hispanic	Not Hispanic	83.7	38362	83.8
	Mexican	9.8	3808	9.7
	Other Hispanic	6.5	2348	6.4
Household Income	$19,999 or less	9.7	8873	9.7
	$20,000–$34,999	10.6	6676	10.6
	$35,000–$49,999	10.9	4921	10.9
	$50,000–$64,999	10.5	4299	10.5
	$65,000–$79,999	9.2	3461	9.1
	$80,000–$99,999	10.4	2968	10.3
	$100,000–$124,999	10.1	3438	10.1
	$125,000–$199,999	14.6	5248	14.6
	$200,000 and above	9.1	2403	9
	Unanswered	4.9	2231	5.2
Race	White	74.1	34155	74.1
	Black	12.1	4984	12.1
	Asian or Pacific Islander	6.9	2155	6.9
	Other race	7	3224	7
Region	Midwest	20.8	9811	20.9
	Northeast	17.4	8508	17.7
	South	37.9	16905	38.2
	West	23.8	9294	23.2
Vote in 2016	Clinton	27.7	13655	27.7
	Trump	26.5	16189	26.5
	Other vote	3.3	2446	3.3
	No vote	42.5	12228	42.5

Online Supplemental Appendix Table 2: Full Regression Model with Confidence Intervals

	Physical or Mental Health Appointment Not Scheduled	Physical or Mental Health Appointment Cancelled or Postponed	Plan to Cancel or Postpone Physical or Mental Health Appointment	Cancer Screening Cancelled or Postponed	Plan to Cancel or Postpone Cancer Screening
Intercept	**2.40 (2.10, 2.73)**	0.85 (0.71, 1.03)	**0.14 (0.10, 0.18)**	**0.06 (0.05, 0.09)**	**0.09 (0.06, 0.12)**
Age 40-64	**0.76 (0.73, 0.79)**	**0.85 (0.80, 0.91)**	**0.72 (0.66, 0.79)**	**1.26 (1.16, 1.38)**	0.85 (0.78, 0.94)
Age 65+	**0.60 (0.57, 0.63)**	0.92 (0.86, 1.00)	**0.54 (0.47, 0.61)**	1.00 (0.89, 1.13)	**0.65 (0.56, 0.75)**
Female	**0.87 (0.84, 0.90)**	1.05 (1.00, 1.10)	**0.63 (0.58, 0.68)**	**1.52 (1.41, 1.64)**	**0.85 (0.78, 0.93)**
Some college	**0.82 (0.79, 0.86)**	**1.11 (1.05, 1.19)**	0.98 (0.88, 1.09)	1.12 (1.01, 1.24)	0.90 (0.81, 1.02)
College and above	**0.67 (0.64, 0.71)**	**1.37 (1.28, 1.47)**	**1.95 (1.75, 2.18)**	**1.73 (1.56, 1.93)**	**1.70 (1.51, 1.92)**
Household income 2nd tercile	0.94 (0.90, 0.99)	0.92 (0.86, 0.99)	0.85 (0.75, 0.96)	0.85 (0.76, 0.96)	0.86 (0.75, 0.98)
Household income 3rd tercile	**0.82 (0.78, 0.86)**	1.12 (1.04, 1.21)	1.10 (0.97, 1.23)	1.20 (1.07, 1.35)	1.05 (0.93, 1.20)
Household income missing	**1.21 (1.11, 1.31)**	0.91 (0.80, 1.04)	1.02 (0.81, 1.29)	0.79 (0.63, 1.01)	0.80 (0.61, 1.05)
Independent	**1.18 (1.13, 1.24)**	0.89 (0.83, 0.96)	**0.63 (0.55, 0.72)**	**0.67 (0.59, 0.76)**	**0.56 (0.49, 0.65)**
Republican	0.98 (0.95, 1.03)	0.95 (0.90, 1.01)	**0.85 (0.78, 0.92)**	1.00 (0.93, 1.09)	**0.83 (0.76, 0.92)**
Tested positive for COVID-19	**0.58 (0.52, 0.65)**	**6.87 (5.86, 8.06)**	**9.37 (7.99, 10.99)**	**9.45 (8.05, 11.09)**	**10.68 (9.04, 12.61)**
Believes had COVID-19	0.87 (0.80, 0.95)	**2.19 (1.95, 2.45)**	**2.36 (2.08, 2.68)**	**3.28 (2.89, 3.72)**	**3.61 (3.18, 4.10)**
Household had COVID-19	0.87 (0.77, 0.99)	**2.03 (1.72, 2.39)**	**1.96 (1.60, 2.40)**	**2.19 (1.77, 2.69)**	**2.69 (2.19, 3.30)**
County COVID-19 deaths, log	0.98 (0.92, 1.03)	1.07 (0.99, 1.15)	1.10 (0.98, 1.23)	1.11 (0.99, 1.24)	0.99 (0.87, 1.13)
Number of health conditions	**0.64 (0.62, 0.65)**	**1.19 (1.16, 1.22)**	**1.12 (1.08, 1.17)**	**1.21 (1.17, 1.26)**	**1.20 (1.15, 1.25)**
Black	0.94 (0.88, 1.00)	0.94 (0.87, 1.02)	1.19 (1.05, 1.36)	0.91 (0.80, 1.03)	1.07 (0.94, 1.23)
Hispanic	0.95 (0.90, 1.00)	1.08 (1.00, 1.16)	**1.26 (1.12, 1.41)**	0.98 (0.87, 1.09)	1.01 (0.90, 1.15)
Asian or Pacific Islander	**1.52 (1.42, 1.63)**	0.95 (0.85, 1.05)	1.12 (0.94, 1.32)	**0.67 (0.56, 0.80)**	1.05 (0.87, 1.26)
Other race	**1.21 (1.10, 1.34)**	1.02 (0.88, 1.18)	0.94 (0.74, 1.20)	0.86 (0.69, 1.08)	0.85 (0.64, 1.11)
Midwest	1.02 (0.97, 1.08)	**0.79 (0.74, 0.86)**	**0.59 (0.52, 0.67)**	**0.81 (0.72, 0.92)**	**0.67 (0.59, 0.77)**
South	1.05 (0.99, 1.10)	**0.75 (0.70. 0.81)**	**0.75 (0.67, 0.83)**	0.93 (0.84, 1.03)	**0.75 (0.66, 0.84)**

	**Physical or Mental Health Appointment Not Scheduled**	**Physical or Mental Health Appointment Cancelled or Postponed**	**Plan to Cancel or Postpone Physical or Mental Health Appointment**	**Cancer Screening Cancelled or Postponed**	**Plan to Cancel or Postpone Cancer Screening**
West	1.09 (1.03, 1.15)	**0.81 (0.75, 0.88)**	0.85 (0.75, 0.95)	0.98 (0.88, 1.10)	0.97 (0.86, 1.10)
Depression mild	0.95 (0.90, 1.00)	**1.16 (1.09, 1.25)**	**1.64 (1.46, 1.83)**	**1.52 (1.37, 1.69)**	**1.61 (1.42, 1.82)**
Depression moderate	0.93 (0.87, 0.99)	**1.41 (1.29, 1.54)**	**1.96 (1.72, 2.24)**	**1.83 (1.61, 2.08)**	**2.35 (2.03, 2.71)**
Depression mod severe	0.92 (0.85, 1.01)	**1.36 (1.21, 1.53)**	**1.80 (1.52, 2.12)**	**1.60 (1.35, 1.89)**	**1.76 (1.46, 2.11)**
Depression severe	0.92 (0.82, 1.03)	**1.33 (1.14, 1.55)**	**1.75 (1.43, 2.14)**	**1.64 (1.33, 2.00)**	**1.66 (1.33, 2.08)**
COVID-19 risk mod low	**0.91 (0.87, 0.95)**	**1.15 (1.08, 1.23)**	0.91 (0.81, 1.01)	1.01 (0.91, 1.12)	0.96 (0.85, 1.08)
COVID-19 risk not high or low	1.03 (0.98, 1.08)	**1.17 (1.10, 1.25)**	0.92 (0.82, 1.03)	0.99 (0.89, 1.10)	0.91 (0.81, 1.03)
COVID-19 risk mod high	**0.84 (0.78, 0.90)**	**1.45 (1.32, 1.58)**	1.05 (0.91, 1.20)	1.20 (1.05, 1.37)	1.00 (0.86, 1.16)
COVID-19 risk very high	0.95 (0.84, 1.07)	**1.37 (1.17, 1.59)**	**1.68 (1.39, 2.03)**	**1.61 (1.33, 1.94)**	**1.76 (1.44, 2.15)**
Anxiety mild	**0.87 (0.83, 0.91)**	**1.28 (1.20, 1.37)**	**1.38 (1.22, 1.55)**	**1.42 (1.28, 1.58)**	**1.41 (1.25, 1.60)**
Anxiety moderate	**0.80 (0.76, 0.85)**	**1.42 (1.32, 1.54)**	**1.78 (1.57, 2.02)**	**1.40 (1.24, 1.58)**	**1.41 (1.23, 1.62)**
Anxiety severe	**0.67 (0.61, 0.74)**	**2.09 (1.84, 2.37)**	**2.56 (2.16, 3.03)**	**2.17 (1.84, 2.57)**	**2.28 (1.90, 2.74)**
Uncertainty tolerance medium	0.96 (0.92, 1.01)	1.08 (1.01, 1.15)	1.07 (0.96, 1.19)	0.93 (0.84, 1.03)	0.91 (0.81, 1.02)
Uncertainty tolerance low	0.91 (0.86. 0.97)	**1.18 (1.09, 1.28)**	**1.25 (1.10, 1.42)**	1.10 (0.98, 1.24)	1.18 (1.03, 1.35)
Coping Approach moderate	**0.92 (0.88, 0.96)**	**1.29 (1.21, 1.37)**	1.14 (1.04, 1.26)	1.14 (1.04, 1.25)	1.02 (0.92, 1.13)
Coping Approach high	**0.80 (0.76, 0.83)**	**1.63 (1.54, 1.73)**	**1.18 (1.07, 1.30)**	1.15 (1.05, 1.25)	1.01 (0.91, 1.12)
Coping Avoid	1.00 (0.96, 1.04)	**1.23 (1.16, 1.29)**	1.03 (0.95, 1.12)	1.06 (0.98, 1.15)	0.97 (0.89, 1.06)
Survey Wave 2	0.97 (0.92, 1.02)	**0.58 (0.54, 0.62)**	**0.75 (0.68, 0.84)**	0.98 (0.88, 1.09)	1.04 (0.91, 1.17)
Survey Wave 3	0.97 (0.92, 1.02)	**0.27 (0.25, 0.29)**	**0.59 (0.53, 0.66)**	**0.82 (0.74, 0.92)**	0.93 (0.82, 1.06)
Survey Wave 4	**0.83 (0.79, 0.88)**	**0.26 (0.24, 0.28)**	**0.54 (0.49, 0.60)**	**0.73 (0.66, 0.81)**	0.88 (0.78, 0.99)

Each column presents odds ratios (95% confidence intervals) from a separate regression model that includes all four survey waves.
For referent categories see Table 1.
N varies by model.
Odds ratio (95% confidence interval in **bold** signifies p<0.001.

Online Supplemental Appendix Figure: Factors associated with Cancelled and Postponed Physical and Mental Health Care and Cancer Screening, all survey waves

[figure]

Coefficient for variable "Tested Positive for COVID" excluded from Figure because its large estimated effect drastically increase scale of x−axis. Regression model includes all survey waves with survey wave as fixed effect.
For referent categories see Table 1.
N varies by model.
Columns 1 and 2 based on responses to two questions about provider and patient cancellations.
"Nothing scheduled" and "Not sure" excluded from analysis for columns 2 – 5.
